# Refining the genetic architecture of myocardial infarction through integrative post-genome-wide association study functional genomics analysis

**DOI:** 10.1093/bioadv/vbag320

**Published:** 2026-10-23

**Authors:** Md Shariful Islam, Md Manir Hossain Mollah, Md Asif Ahsan, Mohammad Ali, Nibas Kumar Pal, Md Fahim Faysal, Md Nurul Haque Mollah

**Affiliations:** Bioinformatics Lab (Dry), Department of Statistics and Data Science, University of Rajshahi, Rajshahi 6205, Bangladesh; Department of Physical Science, Independent University, Bangladesh (IUB), Dhaka 1229, Bangladesh; Bioinformatics Lab (Dry), Department of Statistics and Data Science, University of Rajshahi, Rajshahi 6205, Bangladesh; Bioinformatics Lab (Dry), Department of Statistics and Data Science, University of Rajshahi, Rajshahi 6205, Bangladesh; Bioinformatics Lab (Dry), Department of Statistics and Data Science, University of Rajshahi, Rajshahi 6205, Bangladesh; Department of Computer Science and Engineering, Rajshahi University of Engineering and Technology, Rajshahi 6204, Bangladesh; Bioinformatics Lab (Dry), Department of Statistics and Data Science, University of Rajshahi, Rajshahi 6205, Bangladesh

## Abstract

**Motivation:**

Myocardial infarction is a leading cause of death worldwide and has a strong genetic component. Genome-wide association studies have identified many associated loci, but the genes and variants through which most of them act remain unclear. We aimed to refine the genetic architecture of myocardial infarction by integrating complementary post-association evidence.

**Results:**

A meta-analysis of the Million Veteran Program and UK Biobank (62 129 cases and 828 364 controls) identified 135 genome-wide significant risk loci, five of them potentially novel. Gene-based analysis yielded 333 significant genes, fine-mapping prioritized 613 high-confidence variants, and Bayesian colocalization identified 39 significant gene–tissue signals across 25 eligible loci. Combining all evidence highlighted 10 high-confidence candidate genes, including PHACTR1 and SLC22A3, with enrichment for lipid metabolism, mitogen-activated protein kinase signaling, angiogenesis and chromatin remodeling. Repeating the pipeline with a stricter allele frequency threshold retained 129 loci and full support for 8 of the 10 genes.

**Availability and implementation:**

Input summary statistics are publicly available from the European Bioinformatics Institute genome-wide association study catalog (accessions GCST90475932 and GCST90473536); derived results are provided in the supplementary data.

## 1 Introduction

Myocardial infarction (MI) remains one of the leading causes of morbidity and mortality worldwide and represents a major global public health burden (Salari *et al.* 2023, Thierry *et al.* 2026). MI primarily results from the interruption of coronary blood flow due to atherosclerotic plaque rupture and thrombotic occlusion, leading to ischemic injury and irreversible myocardial damage (Fuster *et al.* 1990, Severino *et al.* 2020). Despite substantial advances in prevention, diagnosis, and treatment, MI continues to contribute significantly to cardiovascular mortality and long-term disability (Salari *et al.* 2023, Zhan *et al.* 2023). Both environmental and genetic factors contribute to MI susceptibility, with heritability estimates suggesting a substantial genetic component underlying disease risk (Bhattacharya *et al.* 2025, Oliveira *et al.* 2015).

Genome-wide association studies (GWAS) have substantially advanced the understanding of the genetic architecture of MI and coronary artery disease (CAD) by identifying numerous susceptibility loci (Hartiala *et al.* 2021, Thierry *et al.* 2026). Large-scale biobank-based studies, including the Million Veteran Program (MVP) and UK Biobank (UKB), have enabled the identification of thousands of genome-wide significant variants through increased sample sizes and improved statistical power (Nikpay *et al.* 2015, Hartiala *et al.* 2021, Tcheandjieu *et al.* 2022). However, the biological interpretation of many GWAS signals remains challenging. Common variants collectively explain only approximately 10%–15% of the estimated heritability of MI (Dikilitas *et al.* 2020), and most GWAS-associated variants are located in non-coding genomic regions, making it difficult to identify the genes through which they may influence disease risk (Al-Ali *et al.* 2023). In addition, linkage disequilibrium (LD) can result in multiple correlated variants within associated regions, complicating the identification of variants most likely to contribute to the observed associations (Aragam *et al.* 2022, D’Antonio *et al.* 2023). These challenges have motivated the use of complementary post-GWAS approaches, including functional mapping, gene-based association analysis, statistical fine-mapping, eQTL integration, and Bayesian colocalization, to improve the functional interpretation and prioritization of disease-associated signals (Giambartolomei *et al.* 2014, Miller *et al.* 2016, Watanabe *et al.* 2017, Aragam *et al.* 2022, Seo *et al.* 2024, Wang *et al.* 2024).

Building on these advances, large-scale GWAS and post-GWAS studies have provided an important foundation for understanding MI and CAD genetic architecture (Nikpay *et al.* 2015, Hartiala *et al.* 2021, Aragam *et al.* 2022, Tcheandjieu *et al.* 2022). However, the strength of evidence supporting individual candidate genes can vary across analytical approaches, highlighting the value of integrating complementary genetic and functional evidence. Although integrative functional-genomics approaches have been applied to cardiovascular traits, including CAD (Miller *et al.* 2016), a convergent framework for MI combining gene-based association, functional mapping, fine-mapping, Bayesian colocalization, and explicit multi-evidence gene prioritization, together with assessment of robustness to variant-frequency filtering, remains less well characterized. In this study, we therefore performed an integrative genomic analysis of MI using harmonized MVP and UKB GWAS summary statistics, combining these complementary approaches to prioritize high-confidence candidate genes and putative causal variants. We further evaluated the robustness of the prioritized candidates using MAF-based sensitivity analysis and characterized the resulting signals through regional association and functional enrichment analysis. This framework was designed to refine established and potentially novel MI-associated signals and distinguish candidates supported by convergent evidence from those requiring further independent and functional validation. Figure 1 displays the workflow of this study.

**Figure 1:**
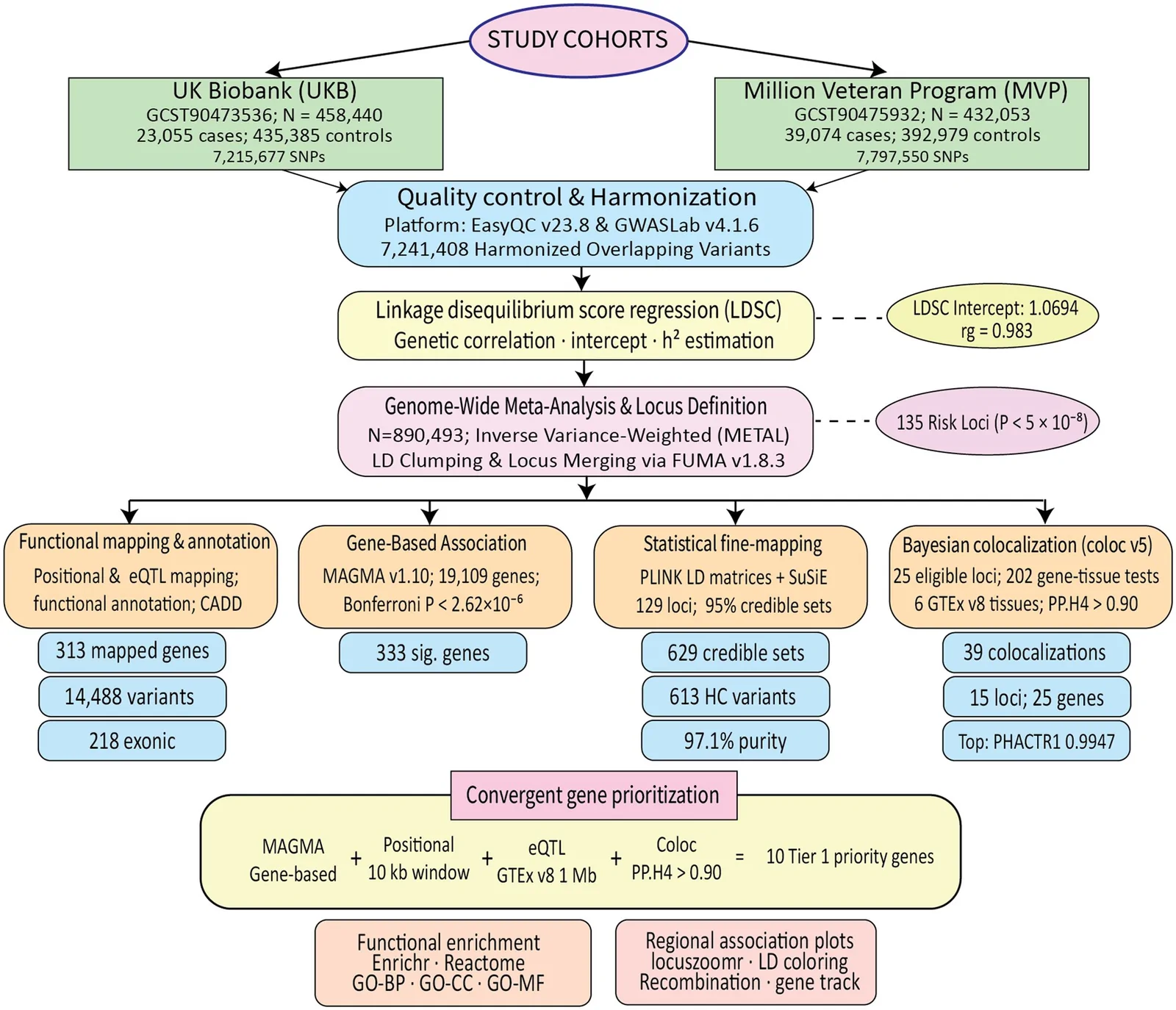
Analytical workflow for the integrative genomic analysis of myocardial infarction.

## 2 Methods

### 2.1 Source of data and study cohorts

For the genome-wide association study (GWAS) analysis of myocardial infarction (MI), we used GWAS summary statistics from two cohorts of European ancestry: the Million Veteran Program (MVP; 39 074 cases and 392 979 controls; total *n* = 432 053) and the UK Biobank (UKB; 23 055 cases and 435 385 controls; total *n* = 458 440). The summary statistics were obtained from the NHGRI-EBI GWAS Catalog, a publicly accessible repository of GWAS summary-level data maintained by the National Human Genome Research Institute and the European Bioinformatics Institute (NHGRI-EBI) (MacArthur *et al.* 2017). The MVP and UKB summary statistics were retrieved using the accession identifiers GCST90475932 (GWAS Catalog 2026a) and GCST90473536 (GWAS Catalog 2026b), respectively. Information on variant level association, such as SNP identifiers, chromosomal positions, effect and non-effect alleles, effect size estimates, allelic frequencies, standard errors and association *P*-values, were included for each summary statistic. The combined meta-analysis comprised 62 129 myocardial infarction cases and 828 364 controls (total *n* = 890 493). A summary of the GWAS datasets included in the meta-analysis is provided in Table 1, available as supplementary data at *Bioinformatics Advances* online.

**Table 1:** High-confidence Tier 1 candidate genes prioritized through integrative genomic analysis of myocardial infarction.^a^^,^^b^

Rank	Gene	Locus	Chr	Lead SNP	MAGMA Z	MAGMA P	Locus PIP	PP.H4	Coloc Tissue
1	*PHACTR1*	47	6	rs9349379	11.72	4.93 × 10^–32^	1.0	0.9947	Artery Coronary
2	*SLC22A3*	57	6	rs140570886	6.11	5.00 × 10^–10^	1.0	0.9653	Artery Aorta
3	*TMEM116*	93	12	rs597808	6.44	5.96 × 10^–11^	1.0	0.9418	Artery Aorta
4	*AIDA*	11	1	rs35700460	6.11	5.00 × 10^–10^	1.0	0.9358	Heart Left Ventricle
5	*CFDP1*	109	16	rs1364077	5.91	1.69 × 10^–09^	1.0	0.9339	Heart Atrial App.
6	*LPL*	66	8	rs2083637	6.79	5.76 × 10^–12^	1.0	0.9267	Whole Blood
7	*MAPKAPK5*	93	12	rs597808	6.29	1.55 × 10^–10^	1.0	0.9261	Artery Aorta
8	*SEMA5A*	41	5	rs17263150	4.95	3.77 × 10^–07^	1.0	0.9155	Artery Aorta
9	*SRR*	113	17	rs7209460	5.50	1.91 × 10^–08^	1.0	0.9149	Liver
10	*MTAP*	71	9	rs1556515	7.11	5.84 × 10^–13^	1.0	0.9068	Artery Aorta

a App., appendage; Chr, chromosome; Coloc, colocalization; MAGMA Z/P, gene-based *Z* statistic and *P*-value; PIP, posterior inclusion probability; PP.H4, posterior probability of a shared causal variant (colocalization hypothesis 4); SNP, single-nucleotide polymorphism.

b All 10 genes achieved the maximum weighted evidence score of 8, indicating support from all four analytical approaches. Shown are genomic loci, lead SNPs, MAGMA statistics, fine-mapping PIPs, Bayesian colocalization probabilities (PP.H4), and the GTEx tissue with the strongest colocalization evidence. Because all Tier 1 genes shared the same weighted score, genes are ranked by PP.H4 in descending order.

### 2.2 Quality control and harmonization of GWAS summary statistics

Quality control of GWAS summary statistics was performed using EasyQC v23.8 (Rangamaran *et al.* 2018) to identify inconsistencies and errors prior to downstream analyses. Variants with missing information, duplicated entries, inconsistent allele annotations, invalid chromosomal coordinates, non-finite or implausible effect-size (β) estimates, invalid *P*-values, and non-finite test statistics were excluded. Effect sizes are reported throughout as signed β coefficients on the log-odds-ratio scale rather than as untransformed odds ratios. Strand-ambiguous variants (A/T and C/G) and variants with minor allele frequency (MAF) < 0.01 were also removed. Allele harmonization between MVP and UKB summary statistics was subsequently performed using GWASLab v4.1.6 (He *et al.* 2023) to ensure consistent variant representation and effect allele orientation, and allele frequency concordance across cohorts. Variants with reversed allele orientation were aligned by allele flipping, while unresolved mismatched variants were excluded from further analyses.

### 2.3 LDSC and genetic correlation analysis

Linkage disequilibrium score regression (LDSC v2.0.1) was performed using LD Score Regression to evaluate the genetic architecture of myocardial infarction (MI) and assess inflation in GWAS summary statistics (Bulik-Sullivan *et al.* 2015b). Analyses were conducted separately for the MVP and UKB datasets using European reference LD scores consistent with the ancestry composition of the cohorts. SNP-based heritability, genomic inflation factors (λGC), LDSC intercepts, and attenuation ratios were estimated to distinguish inflation attributable to polygenicity from potential confounding factors, including population stratification. Genetic correlation analysis between the MVP and UKB GWAS summary statistics was also performed using LDSC regression to evaluate the extent of shared genetic architecture between the two cohorts prior to meta-analysis (Bulik-Sullivan *et al.* 2015a). This analysis was performed on the cohort-specific GWAS summary statistics for MVP and UKB following the quality control and harmonization procedures, but prior to inverse-variance-weighted meta-analysis, ensuring that the genetic correlation estimate reflects the concordance of independently ascertained association signals rather than any shared post-meta-analysis artifact. The observed genetic correlation was used to assess the consistency of association signals and support the suitability of combining the datasets in a subsequent meta-analysis. For the combined meta-analysis, observed-scale SNP heritability was additionally transformed to the liability scale using LDSC, based on the sample prevalence of the meta-analysis (62 129 cases and 828 364 controls) and an assumed myocardial infarction population prevalence of 8.2%, consistent with a recent large-scale European ancestry cardiovascular genetics study, thereby enabling transformation of the observed-scale SNP heritability to the liability scale (Rocheleau *et al.* 2024).

### 2.4 Genome-wide association analysis

Genome-wide association meta-analysis was performed using harmonized GWAS summary statistics from the MVP and UKB cohorts with METAL (v2020-05-05) (Willer *et al.* 2010). Following the quality control and harmonization procedures described in the Section 2.2, only overlapping variants shared between the datasets were retained for downstream analyses. During meta-analysis, genomic control correction (GENOMICCONTROL ON) was applied to account for residual inflation in the study-specific association statistics before combining the datasets. Allele frequencies were averaged across studies (AVERAGEFREQ ON), minimum and maximum allele frequencies were reported (MINMAXFREQ ON), and effective sample size was used for weighting. A fixed-effects inverse-variance-weighted model was used for meta-analysis, as implemented by default in METAL, given the shared European ancestry and comparable phenotype definitions of the two contributing cohorts. MVP and UKB are independently ascertained cohorts, with no known overlapping participants. As an additional assessment of potential inflation attributable to residual confounding, including possible sample overlap, the LDSC intercept was examined. Heterogeneity between the MVP and UKB cohorts was evaluated using the ANALYZE HETEROGENEITY option, which generated Cochran’s Q statistic and the *I^2^* statistic to assess the consistency of genetic effects across studies (Ioannidis *et al.* 2007). Combined association statistics, including pooled effect estimates, standard errors, weighted allele frequencies, and overall association *P*-values for MI, were calculated for each variant. Genome-wide significant variants were identified using the conventional threshold of *P *< 5 × 10^−^^8^ (Fadista *et al.* 2016, Islam *et al.* 2026). The resulting meta-analysis summary statistics were subsequently used for genomic risk locus identification, functional annotation, statistical fine-mapping, Bayesian colocalization analysis, and regional association visualization.

### 2.5 Genomic risk loci identification and functional annotation

Functional mapping and genomic risk locus identification were performed using FUMA (Functional Mapping and Annotation; v1.8.3) (Watanabe *et al.* 2017) based on the meta-analyzed GWAS summary statistics. Genome-wide significant variants passing the threshold of *P *< 5 × 10^−^^8^ were used for downstream annotation and locus definition. Analyses were conducted using the European population reference panel from the 1000 Genomes Project Phase 3 dataset (Auton *et al.* 2015). Within FUMA, independent significant SNPs and lead SNPs were identified using linkage disequilibrium (LD)-based clumping procedures with LD thresholds of r^2^ ≥ 0.6 for independent significant SNPs and r^2^ ≥ 0.1 for lead SNP definition (Machiela and Chanock 2015). Genomic regions located within 250 kb were merged to define genomic risk loci. Functional annotation of associated variants was performed within FUMA to evaluate the potential biological relevance of identified loci. Positional gene mapping was conducted using a 10 kb window around associated variants, while expression quantitative trait locus (eQTL) mapping was performed using a 1 Mb window based on GTEx v8 tissue datasets, including arterial tissues, heart tissues, liver, whole blood, and EBV-transformed lymphocytes. Positional and eQTL mapping incorporated FUMA’s default deleteriousness filters, whereby variants with CADD scores ≥ 12.37 were annotated irrespective of their RegulomeDB score, whereas variants with RegulomeDB score ≤ 7 were annotated irrespective of their CADD score (FUMA v1.8.3 default threshold) (Watanabe *et al.* 2017). Genomic risk loci lacking a previously reported association with MI in the NHGRI-EBI GWAS Catalog were additionally cross-referenced against Open Targets Genetics, FinnGen (data freeze R12), the GWAS Catalog PheWAS variant view, and HeartBioPortal (incorporating CARDIoGRAMplusC4D summary statistics) for previously reported associations with MI, coronary artery disease, or related cardiovascular phenotypes. A formal literature search was also conducted to identify previously reported associations involving the corresponding lead variants or genomic loci. This cross-database and literature assessment was used to evaluate prior reporting of the identified loci and did not constitute independent replication of the present GWAS findings.

### 2.6 Gene-based association and tissue expression analysis

Gene-based association analysis was performed using MAGMA (v1.10) (de Leeuw *et al.* 2015) implemented within FUMA to identify genes significantly associated with MI. Summary statistics from the meta-analyzed GWAS were used as input, and gene-level test statistics were computed by aggregating SNP-level association signals within each gene boundary. A Bonferroni-corrected significance threshold of *P *< 2.62 × 10^−6^ (0.05 divided by 19 109 genes tested) was applied to define genome-wide significant gene-based associations. The European population reference panel from the 1000 Genomes Project Phase 3 dataset was used for LD estimation during gene-based testing. MAGMA gene-property tissue expression analysis was subsequently performed using GTEx v8 tissue-specific gene expression profiles across 53 tissue types to evaluate tissue-level enrichment of MI-associated genes.

### 2.7 Fine-mapping analysis

Fine-mapping analysis was performed to prioritize putative causal variants within genomic risk loci identified through FUMA functional mapping and annotation (Schaid *et al.* 2018). Genomic regions corresponding to the identified loci were extracted from the meta-analyzed GWAS summary statistics for downstream fine-mapping analyses. Linkage disequilibrium (LD) information for each locus was calculated using PLINK (v1.90b7) (Chang *et al.* 2015), based on the European reference population from the 1000 Genomes Project Phase 3 dataset. This reference panel was selected because the meta-analysis combined predominantly European-ancestry GWAS datasets from the Million Veteran Program (MVP) and UK Biobank (UKB), making it an appropriate and widely used external LD reference for summary-statistics-based fine-mapping.

Statistical fine-mapping was performed using the Sum of Single Effects (SuSiE) algorithm (Wang *et al.* 2020, Zou *et al.* 2022), implemented through the susieR R package (v0.14.2) using the summary-statistics interface (susie_rss). SuSiE estimates posterior inclusion probabilities (PIPs) while simultaneously modeling multiple independent association signals within a locus using GWAS summary statistics and an external LD reference, making it well suited for fine-mapping complex GWAS loci exhibiting allelic heterogeneity. PIPs were estimated for individual variants, and 95% credible sets were constructed to prioritize variants most likely to explain the observed association signals. The LD reference panel comprised 503 European individuals from the 1000 Genomes Projects Phase 3 dataset, and the total meta-analysis sample size (*n* = 890 000) was supplied as the effective sample size. The maximum number of causal signals per locus (L = 10), credible set coverage (0.95), and minimum absolute correlation for credible set purity (0.5), and the maximum number of iterations (max_iter = 1000) were retained at the software-recommended default settings (Wang *et al.* 2020, Zou *et al.* 2022), consistent with standard practice in large-scale GWAS fine-mapping studies (Aragam *et al.* 2022). The prior variance was estimated automatically using the default empirical Bayes procedure implemented in susieR. To improve numerical stability during model fitting, LD matrices were regularized by adding 1 × 10^−4^ to the diagonal prior to analysis. Variants with PIP ≥ 0.9 were considered high-confidence putative causal variants. To characterize their potential functional consequences, high-confidence variants were cross-referenced with the FUMA candidate SNP annotation output. Variants overlapping the FUMA candidate SNP set were annotated according to their genomic consequences and Combined Annotation Dependent Depletion (CADD) scores, whereas variants not present in the FUMA candidate SNP output were retained as fine-mapped candidates but were not included in the FUMA-based functional annotation.

### 2.8 Colocalization analysis

Bayesian colocalization analysis was performed using coloc.abf() implemented in coloc package v5 to evaluate whether MI GWAS association signals shared common causal variants with GTEx v8 eQTL signals (Giambartolomei *et al.* 2014, Wallace 2021). Loci were considered eligible if regional GWAS summary statistics were available, overlapping eQTL signals were detected in at least one of six cardiovascular-relevant tissues (aortic artery, coronary artery, heart left ventricle, heart atrial appendage, liver, and whole blood), and at least 20 overlapping SNPs were present between the GWAS and eQTL datasets. This minimum overlap criterion was predefined to ensure an adequately informative set of shared variants for Bayesian colocalization and to avoid inference from very small overlapping SNP sets. Colocalization analysis used the default prior probabilities of association implemented in coloc.abf() (p1 = 1 × 10^−4^, p2 = 1 × 10^−4^, and p12 = 1 × 10^−5^). Posterior probabilities were estimated for five hypotheses: H0 (no association in either dataset), H1 (association with the GWAS trait only), H2 (association with the eQTL trait only), H3 (association with both traits through distinct causal variants), and H4 (association with both traits through a shared causal variant). A PP.H4 > 0.90 threshold was used to define strong evidence of colocalization (Ayalew *et al.* 2026). For loci with multiple significant gene-tissue pairs, the representative signal was selected based on the highest PP.H4 value and cardiovascular tissue relevance.

### 2.9 Integrative gene prioritization

Integrative gene prioritization was performed to identify high-confidence candidate genes across the myocardial infarction (MI)-associated genomic risk loci by integrating four independent analytical approaches: (i) genome-wide significant gene-based association identified by MAGMA (de Leeuw *et al.* 2015) (Bonferroni-corrected *P* < 2.62 × 10–6), (ii) positional gene mapping within a 10 kb window using FUMA (Watanabe *et al.* 2017), (iii) eQTL mapping using GTEx v8 datasets implemented in FUMA, and (iv) Bayesian colocalization analysis demonstrating strong evidence of shared association signals (PP.H4 > 0.90) (Wallace 2021). Each gene was first assigned an evidence-count score (0–4) according to the number of analytical approaches supporting it. Gene prioritization was subsequently performed using a hierarchical classification and ranking framework. To account for the differing evidentiary strength of the four analytical approaches, an ordinal weighted evidence score was calculated by assigning weights 3, 2, 2, and 1 to Bayesian colocalization, MAGMA gene-based association, eQTL mapping, and positional mapping, respectively. This weighting reflects the generally accepted hierarchy of evidence in post-GWAS gene prioritization (Mountjoy *et al.* 2021), whereby Bayesian colocalization provides the strongest support for a shared causal variant, MAGMA and eQTL mapping provide complementary statistical and functional evidence (de Leeuw *et al.* 2015, Watanabe *et al.* 2017), and positional mapping provides supportive evidence based on physical proximity (Watanabe *et al.* 2017). Genes supported by all four analytical approaches (evidence-count score = 4), corresponding to the maximum weighted score of 8, were classified as Tier 1 high-confidence prioritized genes. The remaining genes were classified according to their original evidence-count score (three, two, or one supporting analytical approach), because different combinations of supporting evidence can produce identical weighted scores. The weighted evidence score was therefore used to refine the ranking of genes within each evidence-count tier rather than redefine tier membership. Within each tier, genes were ranked according to weighted evidence score, followed by Bayesian colocalization posterior probability (PP.H4), fine-mapping posterior inclusion probability (PIP), MAGMA Z-statistic, and finally gene symbol (alphabetical order) to ensure a fully deterministic ranking. Fine-mapping information was incorporated throughout the prioritization process to provide additional statistical support for candidate gene prioritization across associated loci.

### 2.10 Regional association visualization

Regional association visualization was performed for prioritized genomic risk loci identified from the GWAS and fine-mapping analyses to examine local association patterns, linkage disequilibrium (LD) structure, recombination rates, and prioritized variants within each locus. Regional association plots were generated using locuszoomr (Lewis and Wang 2025) based on meta-analyzed GWAS summary statistics, LD information, fine-mapping results, and recombination rate data. Linkage disequilibrium information for each locus was calculated using PLINK (v1.90b7) (Chang *et al.* 2015) with the European population reference panel from the 1000 Genomes Project Phase 3 dataset. Recombination rate data were integrated to facilitate visualization of local recombination patterns across genomic regions.

### 2.11 Functional enrichment analysis

Functional enrichment analysis of the prioritized candidate was performed using Enrichr (Kuleshov *et al.* 2016), a comprehensive gene set enrichment analysis web server. The gene list was submitted to three Gene Ontology (GO) databases Biological Process (BP), Cellular Component (CC), and Molecular Function (MF) and the Reactome pathway database (Good *et al.* 2021). Enrichment significance was assessed using Fisher’s exact test, with adjusted *P*-values calculated using the Benjamini-Hochberg false discovery rate correction, and terms with adjusted *P *< .05 were considered statistically significant (Benjamini *et al.* 2001, Pal *et al.* 2026). Significant GO and Reactome terms were evaluated for their biological relevance to MI and used to provide functional context for the prioritized candidate genes.

### 2.12 Sensitivity analysis

To evaluate the robustness of the primary findings to variant frequency, a sensitivity analysis was performed by repeating the complete analytical pipeline after restricting each individual GWAS summary statistics dataset to variants with a minor allele frequency (MAF) ≥ 0.05. The MAF-filtered GWAS summary statistics were re-meta-analyzed using the same analytical framework described in Section 2.4, and the resulting meta-analyzed summary statistics were subsequently used for FUMA functional mapping and annotation, MAGMA gene-based association analysis, and SuSiE fine-mapping using identical software version, analytical parameters, and significance thresholds as described in Sections 2.5–2.7, including the posterior inclusion probability (PIP) ≥ 0.9 criterion for high-confidence fine-mapped variants. Bayesian colocalization analysis was similarly repeated using the same analytical framework described in Section 2.8, including the same six cardiovascular-relevant GTEx v8 tissues, the same approximate ±1000 bp GWAS-eQTL SNP-matching window, the same minimum SNP eligibility criteria (≥20 overlapping GWAS-eQTL SNPs), and the same posterior probability threshold (PP.H4 > 0.90) for strong colocalization evidence. Integrative gene prioritization was subsequently repeated using the identical evidence-count and weighted-scoring framework described in Section 2.9, with genes attaining the maximum evidence-count score (support from all four analytical approaches) classified as Tier 1, consistent with the primary analysis. The robustness of the primary findings was assessed by comparing the primary and MAF-restricted analyses with respect to (i) the number of genomic risk loci identified and genes mapped through positional and eQTL evidence, (ii) the number of MAGMA-significant genes, (iii) the number of loci successfully fine-mapped and the number of high-confidence fine-mapped variants retained, (iv) the number of loci meeting colocalization eligibility criteria and the number of gene-tissue pairs evaluated, and (v) the overlap between the Tier 1 candidate genes identified in each analysis.

A complete summary of the software versions and key analytical parameters used throughout the analytical workflow is provided in Table 2, available as supplementary data at *Bioinformatics Advances* online.

**Table 2:** GO and Reactome pathway enrichment analysis of prioritized myocardial infarction candidate genes.^a^^,^^b^

BPs				
GO_ID	GO function	*P*-value	Adjusted *P*-value	Enriched Genes
GO:0043508	Negative Regulation of JUN Kinase Activity	.002495	.006325	AIDA
GO:0043149	Stress Fiber Assembly	.00526	.007719	PHACTR1
GO:0010744	Positive Regulation of Macrophage Derived Foam Cell Differentiation	.005957	.009065	LPL
GO:0019433	Triglyceride Catabolic Process	.007292	.010119	LPL
GO:0042632	Cholesterol Homeostasis	.009106	.015696	LPL
GO:0045766	Positive Regulation of Angiogenesis	.011729	.020886	SEMA5A
GO:0050729	Positive Regulation of Inflammatory Response	.013616	.022225	LPL
GO:0006338	Chromatin Remodeling	.018592	.049280	CFDP1

CCs				
GO_ID	GO function	*P*-value	Adjusted *P*-value	Enriched Genes
GO:0000228	Nuclear Chromosome	.004842	.007140	CFDP1
GO:0016323	Basolateral Plasma Membrane	.009974	.014935	SLC22A3
GO:0070062	Extracellular Exosome	.011533	.019728	SEMA5A; MTAP
GO:0031966	Mitochondrial Membrane	.012827	.039728	SLC22A3
GO:0005789	Endoplasmic Reticulum Membrane	.033448	.047931	TMEM116
GO:0005634	Nucleus	.038293	.048609	PHACTR1; MAPKAPK5

MFs				
GO_ID	GO function	*P*-value	Adjusted *P*-value	Enriched Genes
GO:0016841	Ammonia-lyase Activity	.002497	.023103	SRR
GO:0008504	Monoamine Transmembrane Transporter Activity	.005985	.024210	SLC22A3
GO:0004465	Lipoprotein Lipase Activity	.006482	.024210	LPL
GO:0004708	MAP Kinase Kinase Activity	.007476	.024210	MAPKAPK5
GO:0004806	Triacylglycerol Lipase Activity	.011443	.024210	LPL
GO:0030215	Semaphorin Receptor Binding	.011443	.024210	SEMA5A
GO:0003779	Actin Binding	.034724	.048524	PHACTR1

Reactome pathways				
ID	Function	*P*-value	Adjusted *P*-value	Enriched Genes
R-HSA-1237112	Methionine Salvage Pathway	.001996	.002561	MTAP
R-HSA-549127	Organic Cation Transport	.003489	.006561	SLC22A3
R-HSA-373755	Semaphorin Interactions	.003754	.009521	SEMA5A
R-HSA-174824	Plasma Lipoprotein Assembly, Remodeling, and Clearance	.004688	.011521	LPL
R-HSA-2559580	Oxidative Stress Induced Senescence	.007821	.017521	MAPKAPK5
R-HSA-5683057	MAPK Family Signaling Cascades	.016390	.03009	MAPKAPK5
R-HSA-1280215	Cytokine Signaling in Immune System	.026870	.039707	MTAP

a GO, Gene Ontology. Adjusted *P*-values were obtained with Benjamini–Hochberg false discovery rate correction.

b Significant enriched biological processes, cellular components, molecular functions, and Reactome pathways identified using Enrichr are presented with adjusted *P*-values and enriched genes.

## 3 Results

### 3.1 GWAS summary statistics and preprocessing

GWAS summary statistics for myocardial infarction (MI) were obtained from the MVP and UKB cohorts and subjected to quality control, filtering, and allele harmonization prior to downstream analyses. Following preprocessing, 7 797 550 variants from MVP and 7 215 677 variants from UKB were retained for analysis. A total of 7 241 408 overlapping variants were subsequently included in the genome-wide association meta-analysis. A summary of preprocessing and harmonization procedures is provided in Table 3, available as supplementary data at *Bioinformatics Advances* online.

### 3.2 LDSC and genetic correlation analysis

Linkage disequilibrium score regression (LDSC) analysis was performed separately for the UKB and MVP GWAS summary statistics to evaluate genomic inflation, SNP-based heritability, and shared genetic architecture between the two cohorts (Table 4, available as supplementary data at *Bioinformatics Advances* online). Following LDSC preprocessing and harmonization with the HapMap3 reference SNPs, 1 144 042 SNPs remained in the UKB dataset, while 1 125 678 SNPs remained in the MVP dataset for downstream LDSC analyses. For the UKB dataset, the mean χ^2^ statistic was 1.29 with a genomic inflation factor (λGC) of 1.209. The observed-scale SNP heritability estimate was 0.029 (SE = 0.0021), and the LDSC intercept was 1.0292 (SE = 0.0103), with an attenuation ratio of 0.1005. The MVP dataset showed a mean χ^2^ statistic of 1.566 and λGC = 1.397, with an observed-scale SNP heritability estimate of 0.042 (SE = 0.0026), an LDSC intercept of 1.1164 (SE = 0.0104), and an attenuation ratio of 0.205. After merging with the European reference linkage disequilibrium panel, 1 137 953 SNPs were retained for genetic correlation analysis, of which 1 119 625 SNPs contained valid alleles. Genetic correlation analysis demonstrated a strong positive genetic correlation (rg) between the UKB and MVP cohorts (rg = 0.9833, SE = 0.0344, 95% CI: 0.9159–1.0507, *P *= 1.3847 × 10^−179^), indicating highly consistent genetic association signals between the two datasets. For the combined meta-analysis, the observed-scale SNP heritability was 0.0349 (SE = 0.0016), corresponding to a liability-scale SNP heritability of 0.1328 (SE = 0.0061) after accounting for the sample prevalence of the meta-analysis and an assumed population prevalence of 8.2%.

### 3.3 Genome-wide association analysis

Genome-wide association analysis integrating the MVP and UKB cohorts was performed using 7 241 408 overlapping variants following harmonization and preprocessing. A total of 14 488 variants surpassed the conventional genome-wide significance threshold of *P *< 5 × 10^−8^. The Manhattan plot demonstrated multiple genome-wide significant association signals distributed across several chromosomes, with particularly prominent loci observed on chromosomes 6 and 9 (Fig. 2). The corresponding quantile-quantile (Q-Q) plot showed clear deviation from the null expectation at the tail of the distribution, consistent with the presence of polygenic association signals (Fig. 1, available as supplementary data at *Bioinformatics Advances* online). The strongest association signal was identified at rs140570886 on chromosome 6 (BP = 160 591 981), with an effect size of -0.4320 (SE = 0.0223) and a *P*-value of 2.73 × 10^−83^. Additional highly significant association signals were identified within chromosome 9 loci, including rs3218020, rs7865618, rs3217991, and rs1556515, all of which showed genome-wide significant association signals in the combined analysis. Chromosome-specific analysis further identified prominent loci across chromosomes 1, 2, 6, 9, 10, 11, 12, 13, 15, and 19, indicating a broad polygenic genetic architecture underlying myocardial infarction.

**Figure 2:**
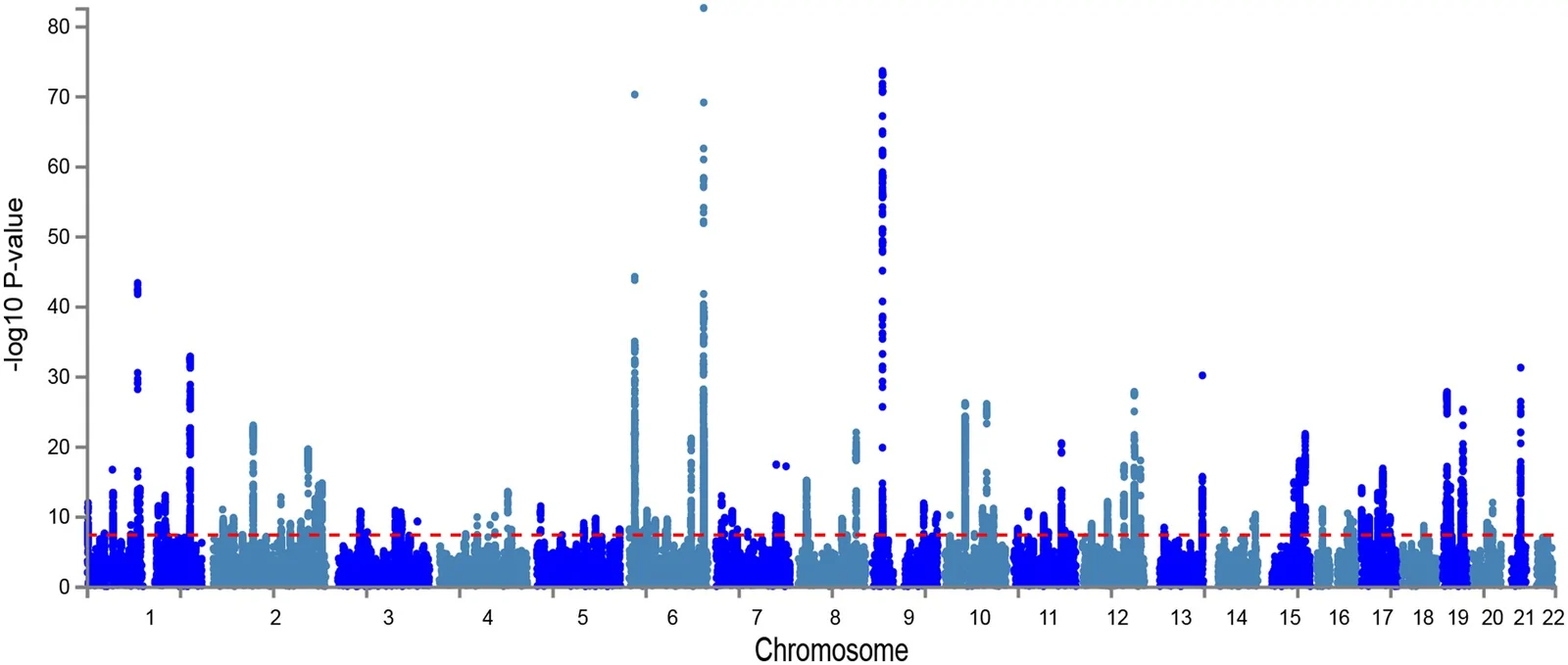
Manhattan plot of the meta-analyzed myocardial infarction GWAS summary statistics. The plot displays genome-wide association signals across chromosomes 1–22, with the *x*-axis representing chromosomal position and the *y*-axis representing − log_10_(*P*-values) for association with myocardial infarction. The dashed horizontal line indicates the genome-wide significance threshold (*P* < 5 × 10^−8^). The combined GWAS had a genomic inflation factor (λGC) of 1.4147 and an LDSC intercept of 1.0694 (SE = 0.0120).

LDSC analysis of the combined GWAS summary statistics demonstrated a genomic inflation factor (λGC) of 1.4147 and a mean χ^2^ statistic of 1.7928. The LDSC intercept was 1.0694 (SE = 0.0120), with an attenuation ratio of 0.087, indicating that most of the observed inflation was attributable to the polygenic genetic architecture of myocardial infarction rather than residual confounding. This degree of genomic inflation is comparable to that reported in large-scale cardiovascular GWAS meta-analyses; e.g. a multi-ancestry CAD GWAS reported λ  =  1.360 and an LDSC intercept of 1.077 (SE = 0.014) in its European-ancestry stratum, with most inflation attributed to polygenicity rather than confounding (Tcheandjieu *et al.* 2022). Heterogeneity analysis demonstrated generally consistent association effects between the MVP and UKB cohorts, with a median I^2^ value of 0 and a mean I^2^ value of 15.79 across analyzed variants. Detailed LDSC metrics for the UKB, MVP, and combined GWAS datasets are provided in Table 4, available as supplementary data at *Bioinformatics Advances* online.

### 3.4 Genomic risk loci identification and functional annotation

Functional mapping and annotation of the meta-analyzed GWAS summary statistics using FUMA (v1.8.3) identified 135 genomic risk loci associated with myocardial infarction (MI) at genome-wide significance (*P *< 5 × 10^−8^) (Table 5, available as supplementary data at *Bioinformatics Advances* online). Comparison with previously reported MI-associated loci through GWAS Catalog interrogation and literature review identified five loci that have not been previously associated with MI, including 2p22.2 (rs7601628), 3p14.1 (rs9845357), 5q21.1 (rs459202), 5q35.3 (rs7719302), and 7q11.22 (rs73154908), representing potentially novel association signals. Independent cross-referencing of these five lead variants against Open Targets Genetics, the FinnGen browser, the GWAS Catalog PheWAS variant view, and HeartBioPortal (incorporating CARDIoGRAMplusC4D) found no previously reported MI or CAD-associated signal in the resources available for each variant (Table 6, available as supplementary data at *Bioinformatics Advances* online). rs9845357 was not evaluable in FinnGen because it did not pass FinnGen-specific genotype quality-control criteria and was therefore assessed using the remaining resources. These findings support their designation as potentially novel rather than previously characterized signals. The 135 loci collectively encompassed 14 488 candidate variants, with association *P*-values ranging from *P *= 4.96 × 10^−8^ to *P *= 2.73 × 10^−83^. The most significant signal mapped to chromosome 6 (index SNP rs140570886; *P *= 2.73 × 10^−83^), followed by the chromosome 9p21 locus (rs3218020; *P *= 2.75 × 10^−74^) and a second chromosome 6 locus (rs9349379; *P *= 6.26 × 10^−71^). LD-based clumping identified 671 independent significant SNPs (r^2^ ≥ 0.6) and 236 lead SNPs (r^2^ ≥ 0.1) across the 135 risk loci (Tables 7 and 8, available as supplementary data at *Bioinformatics Advances* online). Several loci demonstrated substantial allelic heterogeneity; notably, the chromosome 6 locus tagged by rs140570886 contained 87 independent significant SNPs and 26 lead SNPs, the chromosome 9p21 locus (rs3218020) harbored 35 independent significant SNPs, and the chromosome 1 locus tagged by rs17465982 contained 26 independent significant SNPs. Six loci were represented by a single genome-wide significant SNP. Functional consequence annotation of the 14 488 candidate variants revealed that the majority were located within intronic and intergenic regions, followed by ncRNA intronic and untranslated regions. Significant enrichment was observed for intronic, exonic, UTR3, and UTR5 variants relative to the genomic background, whereas intergenic variants were depleted Fig. 3. Detailed functional annotation and enrichment statistics are provided in Table 9, available as supplementary data at *Bioinformatics Advances* online. Among exonic variants, both synonymous and nonsynonymous substitutions were identified, including one stopgain variant (Table 10, available as supplementary data at *Bioinformatics Advances* online).

**Figure 3:**
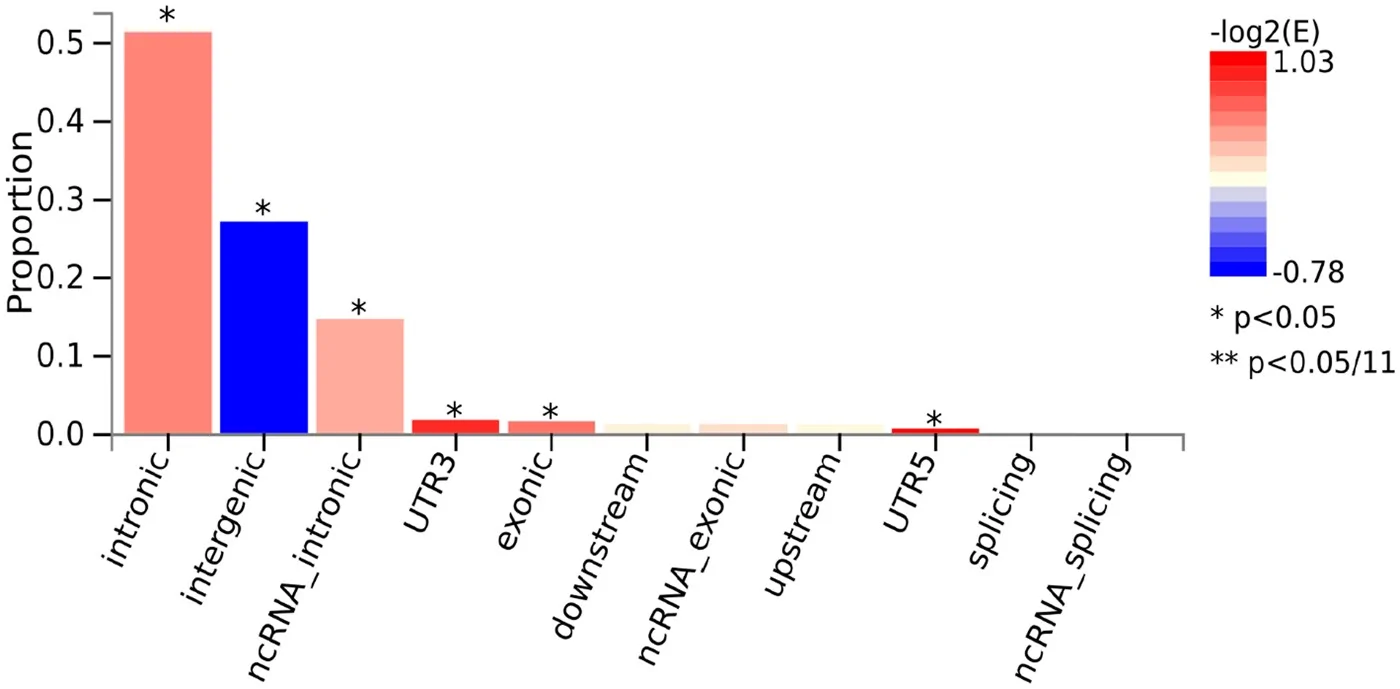
Functional annotation enrichment of genome-wide significant variants identified from the meta-analyzed myocardial infarction GWAS using FUMA. The plot shows the proportion of variants mapped to different functional categories relative to the 1000 Genomes reference panel. Colors represent enrichment values expressed as − log_2_(enrichment), where red indicates enrichment and blue indicate depletion. Asterisks denote statistically significant enrichment after multiple testing correction.

Positional gene mapping within a 10 kb window identified 201 genes across 86 of the 135 risk loci. eQTL mapping using GTEx v8 data spanning aortic artery, coronary artery, heart atrial appendage, heart left ventricle, liver, whole blood, and EBV-transformed lymphocytes identified 209 genes with significant eQTL associations. A total of 97 genes were supported by both positional and eQTL mapping, yielding 313 unique genes across the 135 loci (Fig. 2, available as supplementary data at *Bioinformatics Advances* online). Among positionally mapped variants, the three highest CADD scores were observed for variants within ANGPTL4 (CADD =33.0), ZC3HC1 (CADD = 28.0), and MAPT (CADD = 26.8). Complete positional and eQTL mapping results are provided in Table 11, available as supplementary data at *Bioinformatics Advances* online.

### 3.5 Gene-based association and tissue expression analysis

Gene-based association analysis using MAGMA (v1.10) evaluated 19 109 protein-coding genes across the genome using the combined GWAS summary statistics. Applying a Bonferroni-corrected significance threshold (*P *< 2.62 × 10^−6^), 333 genes reached genome-wide significance (Fig. 3, available as supplementary data at *Bioinformatics Advances* online). The most strongly associated gene was PHACTR1 on chromosome 6 (Z = 11.72, *P *= 4.93 × 10^−32^). Of the 333 significance genes, 186 were corroborated by positional or eQTL mapping in FUMA, of which 76 were supported by both approaches simultaneously, representing the highest level of convergent evidence across all three methods. The remaining 147 MAGMA-significant genes were not identified through positional or eQTL mapping. MAGMA tissue expression analysis using GTEx v8 data demonstrated significant enrichment of MI-associated genes in cardiovascular-related tissues, particularly coronary artery, aortic artery, and heart left ventricle tissues (Fig. 4). A full list of Bonferroni-significant genes with their chromosomal locations, Z-statistics, and mapping status is provided in Table 12, available as supplementary data at *Bioinformatics Advances* online.

**Figure 4:**
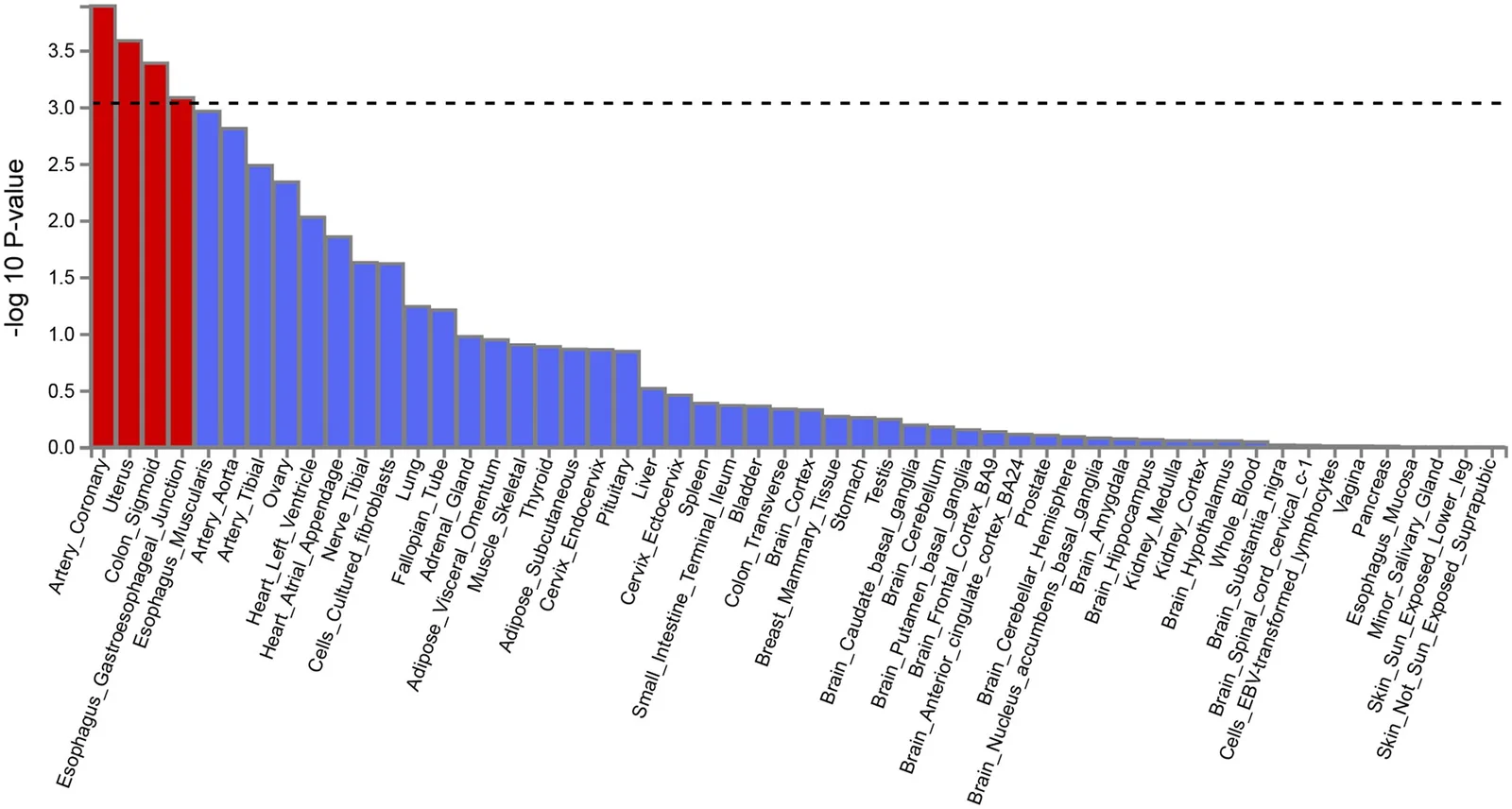
MAGMA tissue expression analysis of myocardial infarction-associated genes using GTEx v8 tissue expression profiles. The plot shows tissue-specific enrichment of gene associations across 53 GTEx tissue types. Cardiovascular-related tissues, including coronary artery, aortic artery, and heart left ventricle, demonstrated significant enrichment after Bonferroni correction. The dashed horizontal line indicates the Bonferroni-corrected significance threshold.

### 3.6 Fine-mapping analysis

SuSiE fine-mapping was performed across 129 of the 135 genomic risk loci, incorporating LD matrices derived from the European 1000 Genomes Project Phase 3 reference panel. The remaining six loci (loci 12, 37, 65, 95, 123, and 128) were excluded as each was represented by a single genome-wide significant variant, precluding LD-based credible set construction. In total, 60 432 variants across the 129 loci were evaluated. Using a 95% credible set coverage, SuSiE identified 629 credible sets across all 129 loci, yielding a mean of 4.9 credible sets per locus (range: 2–10). All 629 credible sets demonstrated high purity, with minimum absolute correlations ≥ 0.80 across all sets (611 of 629 [97.1%] attaining purity = 1.0). Twenty-five loci yielding two credible sets, 20 loci yielded three, and 24 loci yielded four. Ten loci reached the SuSiE maximum of 10 credible sets (32, 43, 54, 66, 71, 74, 98, 105, 118, and 119), indicating extensive allelic heterogeneity. The chromosome 6 locus 57 was resolved to two credible sets despite encompassing 3784 candidate variants. The chromosome 9q21 locus (locus 71) yielded 10 credible sets, all with purity = 1.0.

Applying a PIP ≥ 0.9 threshold, 613 high-confidence putative causal variants were prioritized across all 129 loci, of which 604 (98.5%) attained PIP = 1.0. Among these, 435 overlapped with the FUMA candidate SNP set and were therefore available for functional annotation, including variant consequence and CADD scores, whereas the remaining 178 variants were not present in the FUMA candidate SNP output and therefore were not included in the FUMA-based functional annotation. Among the 435 annotated variants, the majority mapped to intronic (*n* = 212; 48.7%) and intergenic (*n* = 129; 29.7%) regions. Eight variants were exonic, of which seven were nonsynonymous substitutions: rs7412 (APOE, locus 129; CADD = 25.1), rs28929474 (SERPINA1, locus 100; CADD = 20.2), rs11591147 (PCSK9, locus 4; CADD = 15.41), rs60980157 (GPSM1, locus 74; CADD = 14.89), rs17244632 (COBLL1, locus 22; CADD = 14.66), rs1801251 (GIGYF2/KCNJ13, locus 28; CADD = 9.97), and rs2936052 (MIA3, locus 11; CADD = 5.64). The remaining exonic variant, rs3812547 (GPSM1, locus 74; CADD = 7.39), was synonymous. In total, 17 high-confidence variants had CADD scores ≥ 12.37, corresponding to FUMA’s default deleteriousness threshold (Watanabe *et al.* 2017). A complete list of high-confidence fine-mapped variants (PIP ≥ 0.9), together with fine-mapping summary statistics and functional annotations, is provided in Table 13, available as supplementary data at *Bioinformatics Advances* online.

### 3.7 Colocalization analysis

Of the 135 genomic risk loci, 25 satisfied all four eligibility criteria and were taken forward for colocalization analysis. In total, 202 gene-tissue combination tests were conducted across 89 unique genes, spanning six GTEx v8 tissue types: aortic artery, coronary artery, heart atrial appendage, heart left ventricle, liver, and whole blood. Of these, 39 tests (19.3%) yielded strong evidence of colocalization (PP.H4 > 0.90), corresponding to 15 distinct genomic risk loci and 25 unique genes. An additional 58 tests returned PP.H4 values between 0.80 and 0.90, indicating suggestive colocalization, while 28 tests produced PP.H3 ≥ 0.50, suggesting distinct rather than shared causal signals. Significant colocalization signals were distributed across all six tissue types, with aortic artery and heart atrial appendage each contributing eight significant results, followed by heart left ventricle (*n* = 7), whole blood (*n* = 6), liver (*n* = 6), and coronary artery (*n* = 4). The strongest colocalization signal was identified at locus 47, where PHACTR1 demonstrated PP.H4 = 0.9947 in both coronary artery and aortic artery tissue (Fig. 5). Additional high-confidence signals included locus 108 (HPR, heart atrial appendage; PP.H4 = 0.9896), locus 17 (C2orf74, coronary artery; PP.H4 = 0.9838), locus 49 (UHRF1BP1, liver; PP.H4 = 0.9831), and locus 57 (SLC22A3, aortic artery; PP.H4 = 0.9653). Seven loci yielded colocalization signals with more than one gene: locus 29 colocalized with four genes (MST1, IP6K2, NME6, and UBA7), while loci 11, 17, 47, 49, 93, and 108 each colocalized with two or more genes. Complete colocalization results for all 202 gene-tissue tests are provided in Table 14, available as supplementary data at *Bioinformatics Advances* online.

**Figure 5:**
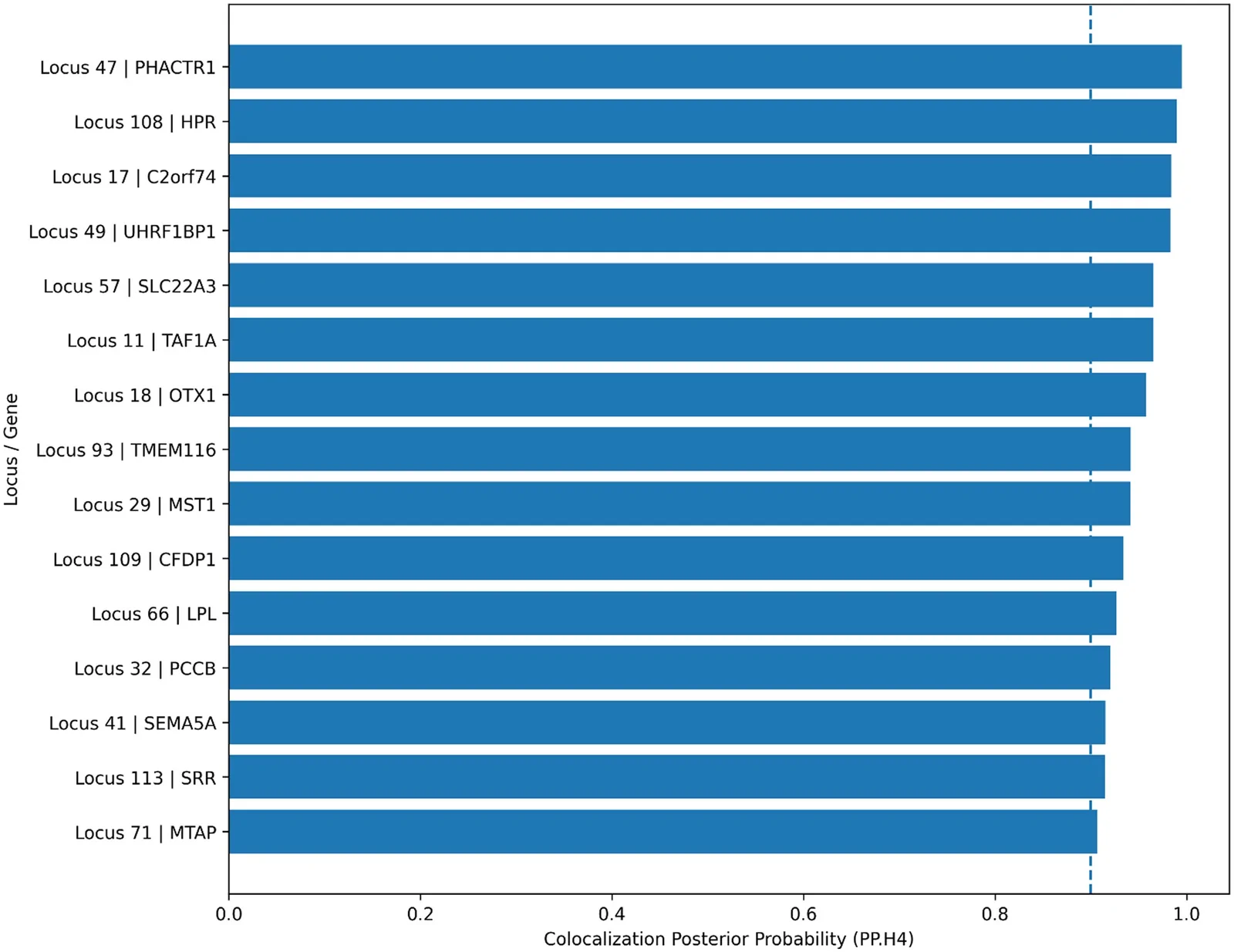
High-confidence GWAS-eQTL colocalization results. Horizontal bar chart showing the best PP.H4 value per locus for the 15 genomic risk loci with at least one gene-tissue colocalization exceeding PP.H4 > 0.90. The dashed vertical line indicates the PP.H4 = 0.90 significance threshold. Gene labels reflect the highest-priority colocalized gene per locus based on PP.H4 magnitude and cardiovascular tissue relevance.

### 3.8 Integrative gene prioritization

A total of 460 unique genes across the 135 genomic risk loci were evaluated using the hierarchical integrative gene prioritization framework. Ten genes were supported by all four analytical approaches, corresponding to the maximum weighted evidence score of 8, and were therefore classified as Tier 1 high-confidence prioritized genes (Table 1). This Tier 1 gene set was identical to that identified under the original evidence-count framework, indicating that incorporation of weighted evidence refined gene ranking without altering the highest-confidence candidate genes. Because all Tier 1 genes shared the maximum weighted score, they were ranked according to Bayesian colocalization posterior probability (PP.H4), followed by fine-mapping posterior inclusion probability (PIP) and MAGMA Z-score where required. The highest-ranked prioritized gene was PHACTR1 (locus 47; MAGMA Z = 11.72; locus PIP = 1.0; PP.H4 = 0.9947 in coronary artery tissue), followed by SLC22A3 (PP.H4 = 0.9653), TMEM116 (PP.H4 = 0.9418), AIDA (PP.H4 = 0.9358), CFDP1 (PP.H4 = 0.9339), LPL (PP.H4 = 0.9267), MAPKAPK5 (PP.H4 = 0.9261), SEMA5A (PP.H4 = 0.9155), SRR (PP.H4 = 0.9149), and MTAP (PP.H4 = 0.9068). All Tier 1 genes exhibited maximal posterior support (PIP =1.0) within their respective fine-mapped loci. An additional 72 genes were supported by three analytical approaches. Among these, 66 genes were supported by MAGMA, positional mapping, and eQTL mapping but were not evaluated in colocalization analyses because of insufficient overlapping eQTL variants within the corresponding loci. Within this tier, genes were ranked using the hierarchical prioritization framework. The highest-ranked Tier 2 genes were TAF1A, MST1, PCCB, C6orf106, and NME6, followed by UHRF1BP1. A further 132 genes were supported by two analytical approaches, whereas 246 genes were supported by a single analytical approach. The complete evidence classification, weighted scores, and final rankings for all 460 prioritized genes are provided in Table 15, available as supplementary data at *Bioinformatics Advances* online.

### 3.9 Regional association plots

Regional association plots were generated for all 10 Tier 1 prioritized genes across nine genomic risk loci (Fig. 4, available as supplementary data at *Bioinformatics Advances* online). The PHACTR1 locus and SLC22A3 locus displayed the strongest association signals (-log_10_*P *= 70.2 and 82.6 respectively), with the SLC22A3 locus showing a complex multi-signal architecture consistent with the 10 credible sets identified by SuSiE. The TMEM116/MAPKAPK5 locus demonstrated a broad association plateau spanning 1.5 Mb, with a recombination peak at ∼111.8 Mb separating the two independently prioritized genes. The AIDA, CFDP1, LPL, and SEMA5A loci each displayed well-resolved signals with LD gradients consistent with their respective fine-mapping credible sets. The SRR locus showed two spatially separated signals clusters reflecting its multi-signal architecture. The MTAP locus exhibited the characteristics bimodal structure of the 9p21 region, with a primary signal at rs1556515 (-log_10_*P *= 73.2) and secondary signals separated by a recombination hotspot at ∼21.95 Mb.

### 3.10 Functional enrichment analysis

Functional enrichment analysis of the 10 Tier 1 prioritized candidate genes was performed using the Enrichr database across Reactome, GO BP, GO CC, and GO MF categories. Several enriched terms remained significant after Benjamini-Hochberg false discovery rate correction (adjusted *P *< .05), and complete results are provided in Table 2. Reactome pathway analysis identified significant enrichment of the Methionine Salvage Pathway (MTAP), Organic Cation Transport (SLC22A3), Semaphorin Interactions (SEMA5A), and Plasma Lipoprotein Assembly, Remodeling, and Clearance (LPL), among others. GO BP enrichment revealed significant terms including Negative Regulation of JUN Kinase Activity (AIDA), Stress Fiber Assembly (PHACTR1), Triglyceride Catabolic Process and Cholesterol Homeostasis (LPL), and Positive Regulation of Angiogenesis (SEMA5A). GO CC analysis showed enrichment in Nuclear Chromosome (CFDP1), Basolateral Plasma Membrane (SLC22A3), Extracellular Exosome (SEMA5A; MTAP), and Endoplasmic Reticulum Membrane (TMEM116). GO MF enrichment identified Lipoprotein Lipase Activity (LPL), MAP Kinase Kinase Activity (MAPKAPK5), Monoamine Transmembrane Transporter Activity (SLC22A3), Ammonia-lyase Activity (SRR), and Actin Binding (PHACTR1) as significant functional annotations.

### 3.11 Sensitivity analysis

To evaluate the robustness of the primary findings to variant frequency, the complete analytical pipeline was repeated after restricting each individual GWAS summary statistics dataset to variants with a minor allele frequency (MAF) ≥ 0.05, followed by re-meta-analysis and downstream analyses. The MAF-restricted analysis identified 129 genomic risk loci, compared with 135 in the primary analysis (Table 16, available as supplementary data at *Bioinformatics Advances* online). FUMA positional and eQTL mapping identified 309 candidate genes under this filter, compared with 313 in the primary analysis (Table 17, available as supplementary data at *Bioinformatics Advances* online); and MAGMA gene-based association identified 317 Bonferroni-significant genes (*P *< 2.62 × 10^−6^), compared with 333 in the primary analysis (Table 18, available as supplementary data at *Bioinformatics Advances* online). Of the 129 identified loci, 124 (96.1%) were successfully fine-mapped, yielding 610 credible sets (97.0% of the 629 credible sets identified in the primary analysis). A total of 560 high-confidence fine-mapped variants (PIP ≥ 0.9) were identified, representing 91.4% of the 613 variants detected in the primary analysis, of which 538 (96.1%) exhibited PIP = 1.0 (Table 19, available as supplementary data at *Bioinformatics Advances* online). Bayesian colocalization analysis, performed using the same analytical framework as the primary analysis, identified 23 loci meeting the predefined eligibility criteria, compared with 25 loci in the primary analysis, resulting in 183 gene-tissue pairs evaluated versus 202 previously (Table 20, available as supplementary data at *Bioinformatics Advances* online).

Integrative gene prioritization based on the updated fine-mapping and colocalization results, evaluated across 318 genes (compared with 460 in the primary analysis), identified 12 genes attaining the maximum evidence score, compared with 10 in the primary analysis (Table 21, available as supplementary data at *Bioinformatics Advances* online). Eight of the ten primary Tier 1 genes (AIDA, CFDP1, MAPKAPK5, MTAP, PHACTR1, SEMA5A, SRR, and TMEM116) retained Tier 1 status under the MAF- restricted analysis, with each maintaining strong colocalization support (PP.H4 > 0.90). In contrast, LPL and SLC22A3 no longer met the colocalization criterion, with PP.H4 decreasing from 0.9267 to 0.2060 and from 0.9653 to 0.4636, respectively. Both genes retained significant MAGMA associations together with positional and eQTL mapping evidence and were therefore reclassified from Tier 1 to an evidence-count score of 3. Conversely, four additional genes (AMT, CDC25A, SNRPC, and TOM1L2) achieved the maximum evidence score exclusively in the MAF-restricted analysis. Complete sensitivity analysis results, including locus, variant, and gene-level comparisons between the primary and MAF-restricted analyses, are provided in Tables 16–21, available as supplementary data at *Bioinformatics Advances* online.

## 4 Discussion

This study performed a large-scale integrative genomic analysis of myocardial infarction (MI) through meta-analysis of MVP and UKB GWAS summary statistics, identifying 135 genomic risk loci, including five potentially novel loci not previously associated with MI (Table 5, available as supplementary data at *Bioinformatics Advances* online). Fine-mapping prioritized 613 high-confidence putative causal variants, while a convergent multi-evidence framework highlighted 10 high-confidence Tier 1 candidate genes. Together, these findings advance the post-GWAS characterization of MI genetic architecture and provide biological insights into the mechanisms underlying identified risk loci. It is important to distinguish these two sets of findings: none of the potentially novel loci contributed a Tier 1 gene, and all 10 Tier 1 genes map to loci that are already well-established MI/CAD signals in the literature. The integrative prioritization framework therefore primarily provides convergent orthogonal evidence for candidate genes at established loci, whereas the potentially novel loci represent a separate, less-characterized set of findings that will require independent validation before their relevance to MI can be established. Notably, the five potentially novel loci were not carried through fine-mapping, colocalization, or gene prioritization, so no causal variant or effector gene is proposed for them here; they are reported solely as previously unreported association signals requiring independent validation. Their apparent novelty should also be interpreted cautiously, as UK Biobank has contributed to many prior GWAS in the GWAS Catalog, and an absent prior association may simply reflect earlier reporting thresholds rather than a truly untested signal.

Functional annotation of the 14 488 genome-wide significant variants revealed that the majority mapped to intronic (51.2%) and intergenic (27.1%) regions, with significant enrichment in intronic (enrichment = 1.41; *P *= 4.56 × 10^−288^), UTR3 (enrichment = 1.81; *P *= 1.24 × 10^−17^), UTR5 (enrichment = 2.03; *P *= 4.85 × 10^−9^), and exonic (enrichment = 1.48; *P *= 4.03 × 10^−8^) regions (Table 9, available as supplementary data at *Bioinformatics Advances* online). This distribution is consistent with prior observations that most GWAS signals for complex traits reside in non-coding regulatory elements rather than protein-coding sequences, underscoring the importance of post-GWAS functional annotation for biological interpretation (Maurano *et al.* 2012, Watanabe *et al.* 2017). Of 218 exonic variants, 92 were nonsynonymous and one was stopgain variant, with the highest CADD scores observed for variants in APOE (rs7412; CADD = 25.1) and PCSK9 (rs11591147; CADD = 15.41), genes encoding well-established regulators of LDL cholesterol metabolism and atherosclerosis risk (Sabatine *et al.* 2017). SuSiE fine-mapping resolved 629 credible sets across 129 loci, with 97.1% attaining maximum purity, and identified 613 high-confidence putative causal variants with PIP ≥ 0.9 (Table 13, available as supplementary data at *Bioinformatics Advances* online). Ten loci reached the SuSiE maximum of 10 credible sets, reflecting substantial allelic heterogeneity consistent with prior reports of complex multi-signal architecture at established coronary artery disease (CAD) and MI-associated loci (Schaid *et al.* 2018). Fine-mapping approaches that compute PIPs are increasingly recognized as critical steps in post-GWAS analysis, substantially narrowing the variant search space for functional follow-up (Schaid *et al.* 2018). In the present study, fine-mapping further prioritized several coding and high-CADD variants, including nonsynonymous and stopgain substitutions, highlighting potentially functional variants underlying MI susceptibility. These findings demonstrate the utility of fine-mapping for refining association signals and prioritizing potentially functional variants within MI-associated loci.

The convergent evidence framework integrating MAGMA gene-based association, positional mapping, eQTL mapping, and Bayesian colocalization identified 10 Tier 1 candidate genes supported by all four independent lines of evidence. This multi-evidence approach is consistent with recommendations for post-GWAS gene prioritization advocating frameworks that combine orthogonal sources of evidence to reduce false positive inherent in any single analytical method (de Leeuw *et al.* 2015, Forgetta *et al.* 2022). The top-ranked gene, PHACTR1 (MAGMA Z = 11.72; PP.H4 = 0.9947 in coronary artery), encodes phosphatase and actin regulator 1. The index SNP rs9349379 (PIP = 1.0) disrupts a MEF2 transcription factor binding site and is a well-established cis-eQTL for PHACTR1 in human coronary arteries, with eQTL effects restricted to arterial tissue in GTEx v8, implicating vascular-specific transcriptional regulation (Codina-Fauteux *et al.* 2018, Jiang *et al.* 2023). Functional studies have demonstrated roles of PHACTR1 in endothelial cell activation and actin stress fiber assembly, consistent with the GO BP enrichment for stress fiber assembly observed in our analysis (Ma *et al.* 2022).

LPL (PP.H4 = 0.9267 in whole blood) encodes lipoprotein lipase, which catalyzes the rate-limiting step in clearance of triglyceride-rich lipoproteins. Human genetic evidence has established a causal link between impaired LPL-mediated lipolysis and CAD risk, with gain-of-function and loss-of-function variants in LPL and its regulators demonstrating concordant effects on triglycerides and CAD risk (Khera and Kathiresan 2017). The enrichment of terms related to plasma lipoprotein remodeling, triglyceride catabolism, cholesterol homeostasis, and macrophage-derived foam cell differentiation provides supportive biological context for the potential involvement of LPL in atherogenesis. MTAP at the 9p21.3 locus (PP.H4 = 0.9068 in aortic artery), one of the strongest and most replicated CAD and MI-associated GWAS signals, has recently been nominated as the effector gene at this locus through CRISPRi tiling integrated with fine-mapping across >500 000 individuals (Audam *et al.* 2025, McPherson and Tybjaerg-Hansen 2016). MTAP loss has been shown to induce pro-fibrotic and angiogenic programs in vascular cells under inflammatory stress. SEMA5A (PP.H4 = 0.9155 in aortic artery) encodes semaphorin 5A, the only class 5 semaphorin with established proangiogenic activity, promoting endothelial cell proliferation and migration through Akt signaling and MMP9-mediated matrix degradation (Sadanandam *et al.* 2010). The GO enrichment for positive regulation of angiogenesis and semaphorin receptor binding provides supportive biological context for the prioritization of SEMA5A at the 5p15.3 locus.

MAPKAPK5, a substrate of the p38 MAPK and ERK3/ERK4 pathways (PP.H4 = 0.9261 in aortic artery), was implicated through Reactome enrichment of MAPK family signaling cascades and oxidative stress-induced senescence. Consistent with this, MAPK signaling more broadly is established in cardiovascular disease pathogenesis, including atherosclerosis and myocardial ischemia-reperfusion injury, through regulation of inflammatory cytokine production and cardiomyocyte apoptosis (Rose *et al.* 2010, Wang *et al.* 2025), supporting a plausible role for MAPKAPK5 within this pathway rather than direct evidence of its own causal contribution. CFDP1 (PP.H4 = 0.9339 in heart atrial appendage) encodes a chromatin remodeling protein that interacts with the SRCAP complex to catalyze H2A.Z deposition, with knockdown disrupting chromosome organization and cell cycle progression in human cells (Messina *et al.* 2017). CFDP1 has been implicated in CAD through large-scale human genetic studies, and our GO CC enrichment for nuclear chromosome and GO BP enrichment for chromatin remodeling provide supportive context for a potential epigenetic regulatory mechanism that warrants further experimental validation. AIDA (PP.H4 = 0.9358 in the heart’s left ventricle) was prioritized as an MI candidate gene through integrative analysis of vascular endothelial cell genomic features; prior functional work has shown that AIDA negatively regulates JNK kinase activity in endothelial cells, a pro-inflammatory and pro-apoptotic pathway implicated in atherosclerosis (Muslin 2008, Lalonde *et al.* 2019), consistent with a possible functional role for AIDA at this locus. SLC22A3 (PP.H4 = 0.9653 in aortic artery) encodes organic cation transporter 3 and has been prioritized as a candidate effector gene at cardiovascular disease-associated loci in multiple post-GWAS analyses, with variants significantly associated with lipoprotein(a) concentration and cardiovascular disease events in familial hypercholesterolemia (Erdmann *et al.* 2018, Paquette *et al.* 2019). SRR (PP.H4 = 0.9149 in liver) contributes to methionine and amino acid metabolic pathways, with growing evidence linking amino acid metabolism to cardiovascular risk through metabolic and inflammatory mechanisms (Bhatt *et al.* 2019).

To assess the extent to which the primary findings depend on the MAF ≥ 0.01 inclusion threshold, we repeated the full analytical pipeline using a more stringent MAF threshold of ≥ 0.05. Eight of the ten Tier 1 genes including PHACTR1, the strongest signal in the study, retained full support across all four analytical approaches under this stricter filter, indicating substantial robustness of the core high-confidence findings to variant-frequency restriction. Two genes, LPL and SLC22A3, no longer met the colocalization threshold when variants with MAF 0.01–0.05 were excluded, despite retaining significant MAGMA, positional, and eQTL evidence. LPL is among the most extensively replicated genes in the coronary artery disease and lipid metabolism literature (Khera and Kathiresan 2017); therefore, the loss of colocalization support under the stricter MAF threshold should be interpreted cautiously and may reflect changes in the available variant set and statistical evidence for colocalization rather than evidence against its previously established biological relevance. SLC22A3 has comparatively less independent replication, and its sensitivity to the MAF threshold therefore warrants further validation. Conversely, four genes (AMT, CDC25A, SNRPC, and TOM1L2) reached the maximum evidence score only under the MAF-restricted analysis. These changes may reflect differences in the variant composition and statistical evidence available under the more stringent filtering criterion, but their biological significance cannot be established from the sensitivity analysis alone. Taken together, these findings support the overall robustness of the core Tier 1 gene set while demonstrating that colocalization evidence can be sensitive to variant-frequency inclusion criteria. They further reinforce the distinction between convergent computational evidence and definitive biological causality, with genes showing MAF-dependent changes requiring independent genetic and functional validation.

Several limitations should be acknowledged. The analysis was restricted to individuals of European ancestry, and studies in diverse populations are needed to evaluate the generalizability of the findings. The present study focused on integrative analysis of publicly available MVP and UKB GWAS summary statistics; consequently, independent replication was not performed within the current analysis, and the identified loci and the 10 Tier 1 candidate genes warrant validation in independent cohorts. Fine-mapping relied on an external European 1000 Genomes Project Phase 3 LD reference panel rather than in-sample LD, which may introduce differences in LD estimation and credible-set resolution. This reference panel (*n* = 503) is substantially smaller than the discovery sample (*n* = 890 493), which may reduce LD estimation precision, particularly for lower-frequency variants; therefore, the 613 high-confidence fine-mapped variants should be regarded as provisional, pending confirmation using larger or more closely matched LD resources, including in-sample LD matrices (e.g. from UK Biobank) where available. Although the LDSC intercept indicated only modest residual inflation beyond polygenicity, substantial confounding is unlikely to have materially biased the genome-wide association statistics; however, subtle locus-specific inflation not captured by a single genome-wide LDSC intercept cannot be fully excluded in downstream fine-mapping and colocalization analyses. Colocalization relied on GTEx v8 bulk-tissue eQTL data, which may not fully capture cell-type-specific regulatory mechanisms relevant to MI, and PP.H4 estimates remain sensitive to prior specification. Although default coloc.abf() priors were used throughout, PP.H4 values near the classification threshold could change under alternative priors. The PP.H4 > 0.90 threshold used here is more stringent than the >0.80 threshold commonly applied in the literature and therefore provides a more conservative criterion, although it does not eliminate dependence on prior specification. Finally, functional enrichment analysis was based on only 10 Tier 1 candidate genes, which may limit the stability of enrichment estimates; terms supported predominantly by individual genes should therefore be interpreted as supportive biological context rather than definitive evidence of pathway involvement. Further independent and experimental studies are required to validate the prioritized candidates and clarify their functional roles.

This *in-silico* integrative genomic study identified 135 MI risk loci and prioritized 10 high-confidence Tier 1 candidate genes whose implicated biological mechanisms span lipid metabolism (LPL), endothelial cytoskeletal regulation (PHACTR1), vascular angiogenesis (SEMA5A), inflammatory signaling (MAPKAPK5 and MTAP), chromatin remodeling (CFDP1), stress kinase regulation (AIDA), organic cation transport (SLC22A3), and amino acid metabolism (SRR). These findings provide a foundation for future functional investigation and experimental validation of candidate genes associated with myocardial infarction.

## 5 Conclusion

This study applied a convergent, multi-evidence post-GWAS framework to harmonized MI GWAS summary statistics, integrating meta-analysis, statistical fine-mapping, functional annotation, and Bayesian colocalization. This approach identified 135 genome-wide significant risk loci, including five potentially novel loci, and prioritized 10 high-confidence Tier 1 candidate genes involved in lipid metabolism, vascular regulation, inflammatory signaling, endothelial function, and chromatin remodeling, with robustness supported by a MAF-based sensitivity analysis. As the Tier 1 genes map to already well-established MI/CAD loci, these findings support the validity of the integrative framework, while the potentially novel loci represent additional signals that will require independent validation. Together, these results refine the genetic architecture of myocardial infarction and provide a foundation for future functional and mechanistic studies of disease susceptibility.

## Supplementary Material

vbag320_Supplementary_Data

## Data Availability

The data underlying this article are available in the GWAS Catalog at https://www.ebi.ac.uk/gwas/studies/GCST90475932 (Million Veteran Program) and https://www.ebi.ac.uk/gwas/studies/GCST90473536 (UK Biobank), and can be accessed with the accession numbers GCST90475932 and GCST90473536, respectively.
