# Supplementary material for: Refining the genetic architecture of myocardial infarction through integrative post-genome-wide association study functional genomics analysis
Source: Bioinform Adv. 2026 Oct 23;6(1):vbag320. doi: 10.1093/bioadv/vbag320 (PMC13730272; doi:10.1093/bioadv/vbag320)
Supplement: Supplementary file 1 [file vbag320_supplementary_data.zip › Supplementary file 2.docx]

**Refining the Genetic Architecture of Myocardial Infarction Through Integrative Post-GWAS Functional Genomics Analysis**

**Md. Shariful Islam^1^,** Md. Manir Hossain Mollah^2^, Md. Asif Ahsan^1^, Mohammad Ali^1^, Nibas Kumar Pal^1^, Md. Fahim Faysal^3^, and Md. Nurul Haque Mollah^1^*

^1^Bioinformatics Lab (Dry), Department of Statistics and Data Science, University of Rajshahi, Rajshahi-6205, Bangladesh.

^2^Department of Physical Science, Independent University, Bangladesh (IUB), Dhaka, Bangladesh

^3^Department of Computer Science and Engineering, Rajshahi University of Engineering and Technology, Rajshahi-6204, Bangladesh

***Corresponding author**: Md. Nurul Haque Mollah (Email: [mollah.stat.bio@ru.ac.bd](mailto:mollah.stat.bio@ru.ac.bd))

**Supporting Items/Captions**

**Supplementary Figures**

**Figure S1:** Quantile-quantile (Q-Q) plot of the meta-analyzed myocardial infarction GWAS summary statistics. The plot compares the observed versus expected -log_10_(*P*-values) under the null hypothesis of no association. Deviation of the observed distribution from the expected distribution at the tail indicates the presence of true association signals for myocardial infarction.

**Figure S2.** Characteristics of the 135 genomic risk loci identified from the meta-analyzed myocardial infarction GWAS using FUMA. For each genomic locus, the plot summarizes locus size (kb), number of associated SNPs, number of mapped genes, and number of genes physically located within the locus boundaries. Substantial variability in locus size, SNP density, and gene content was observed across the identified myocardial infarction risk loci.

**Figure S3.** Annotated Manhattan plot of the meta-analyzed myocardial infarction GWAS summary statistics. Genome-wide significant loci associated with myocardial infarction are shown across chromosomes 1-22, with selected candidate genes annotated at lead association signals. The x-axis represents chromosomal position and the y-axis represents -log_10_(*P*-values). The red dashed horizontal line indicates the genome-wide significance threshold (*P* < 5 × 10^-8^).

**Figure S4.** Regional association plots of prioritized myocardial infarction susceptibility loci. Regional association plots are shown for representative high-confidence loci including AIDA (locus 11), SEMA5A (locus 41), PHACTR1 (locus 47), SLC22A3 (locus 57), LPL (locus 66), MTAP (locus 71), MAPKAPK5 and TMEM116 (locus 93), CFDP1 (locus 109), and SRR (locus 113). For each locus, the upper panel displays association signals as -log_10_(P-values) across the genomic region, with variants colored according to linkage disequilibrium (LD; r²) relative to the lead SNP (purple diamond). The middle panel shows local recombination rates, and the lower panel indicates gene annotations within the corresponding genomic interval. The dashed horizontal line represents the genome-wide significance threshold (P < 5 × 10^-8^).

**Supplementary Figures**

**Figure S1.** Quantile-quantile (Q-Q) plot of the meta-analyzed myocardial infarction GWAS summary statistics. The plot compares the observed versus expected -log_10_(*P*-values) under the null hypothesis of no association. Deviation of the observed distribution from the expected distribution at the tail indicates the presence of true association signals for myocardial infarction.

**Figure S2.** Characteristics of the 135 genomic risk loci identified from the meta-analyzed myocardial infarction GWAS using FUMA. For each genomic locus, the plot summarizes locus size (kb), number of associated SNPs, number of mapped genes, and number of genes physically located within the locus boundaries. Substantial variability in locus size, SNP density, and gene content was observed across the identified myocardial infarction risk loci.

**Figure S3.** Annotated Manhattan plot of the meta-analyzed myocardial infarction GWAS summary statistics. Genome-wide significant loci associated with myocardial infarction are shown across chromosomes 1-22, with selected candidate genes annotated at lead association signals. The x-axis represents chromosomal position and the y-axis represents -log_10_(*P*-values). The red dashed horizontal line indicates the genome-wide significance threshold (*P* < 5 × 10^-8^).

**Figure S4.** Regional association plots of prioritized myocardial infarction susceptibility loci. Regional association plots are shown for representative high-confidence loci including AIDA (locus 11), SEMA5A (locus 41), PHACTR1 (locus 47), SLC22A3 (locus 57), LPL (locus 66), MTAP (locus 71), MAPKAPK5 and TMEM116 (locus 93), CFDP1 (locus 109), and SRR (locus 113). For each locus, the upper panel displays association signals as -log_10_(P-values) across the genomic region, with variants colored according to linkage disequilibrium (LD; r²) relative to the lead SNP (purple diamond). The middle panel shows local recombination rates, and the lower panel indicates gene annotations within the corresponding genomic interval. The dashed horizontal line represents the genome-wide significance threshold (P < 5 × 10^-8^).
